# Disaster preparedness and its correlates among nurses in border areas of Southwest China: A multicenter cross-sectional survey

**DOI:** 10.3934/publichealth.2026039

**Published:** 2026-07-07

**Authors:** Aoli Li, Lihan Chen, Maosong Li, Qiu Ma, Qilian He, Lorna Kwai Ping Suen

**Affiliations:** 1 School of Nursing, Dali University, Dali 671000, China; 2 Department of Nursing, Xianyang Central Hospital, Xianyang 712000, China; 3 Bureau of Science and Technology of Dali Bai Autonomous Prefecture, Dali 671000, China; 4 School of Nursing, Tung Wah College, Hong Kong Special Administrative Region, China

**Keywords:** disaster preparedness, nurses, border areas, resilience, random forest

## Abstract

**Background:**

Nurses play a critical role in disaster preparedness and response, particularly in disaster-prone border areas where health systems may face compounded challenges related to natural hazards, population mobility, and resource constraints. However, empirical evidence on nurses' disaster preparedness in such settings remains limited.

**Objective:**

We aimed to examine disaster preparedness among nurses working in disaster-prone border areas of Southwest China and to identify priority correlates to inform workforce development and training strategies.

**Methods:**

We conducted a multicenter cross-sectional survey of 1366 nurses using multistage stratified cluster sampling in a representative disaster-prone border area of Southwest China (Yunnan Province, China, bordering Myanmar). Data were collected using a general information questionnaire, the Chinese version of the Disaster Preparedness Evaluation Tool, the Resilience Scale of Medical Staff, and the Sense of Professional Mission Scale. Data were analyzed using descriptive statistics, univariate analysis, Pearson correlation analysis, LASSO regression, multivariable linear regression, and random forest analysis.

**Results:**

The mean total disaster preparedness score was 182.32 ± 43.51, indicating a moderate level of preparedness. Random forest analysis identified resilience, gender, and sense of professional mission as the most prominent correlates. In the multivariable linear regression model, resilience, sense of professional mission, gender, and disaster-related training remained significant predictors of disaster preparedness.

**Conclusions:**

Disaster preparedness among nurses working in disaster-prone border areas of Southwest China was moderate. Resilience and sense of professional mission were identified as important correlates of preparedness, highlighting potential priorities for workforce training and public health capacity building in border-area contexts.

## 1. Introduction

Disaster-prone border areas present important public health challenges because of recurrent natural hazards, population mobility, and comparatively constrained health-system capacity. In such settings, frontline health workers must respond not only to acute disaster-related injuries but also to disruptions in routine care and increased service demands during emergencies. Strengthening workforce preparedness in these contexts is therefore an important component of regional health security and health-system resilience.

The frequency and intensity of natural disasters [1] and public health emergencies [2] have increased worldwide in recent years. Disaster events affect human life and health, the environment, infrastructure, and health-system resources. China remains one of the countries frequently affected by natural disasters in Asia [3], and regional disparities in health resources and emergency response capacity continue to shape local preparedness needs. In this context, evidence on disaster preparedness among nurses working in border areas is important for understanding workforce readiness in settings where disaster exposure, population mobility, and resource constraints may coexist.

By the end of 2022, the total number of registered nurses in China had exceeded 5.2 million [4]. As the largest group of health professionals, nurses play essential roles across the mitigation, preparedness, response, and recovery phases of disasters [5]–[7]. Disaster preparedness generally refers to the knowledge, skills, and capacities required to respond effectively to disasters [8]. The level of disaster preparedness among nurses is closely related to the quality and efficiency of emergency response [9] and is therefore of substantial relevance to disaster nursing and public health preparedness.

From a public health perspective, nurses' disaster preparedness is not only an individual competency issue but also an important component of health-system resilience. In border areas of Southwest China, health services may face the combined pressures of recurrent natural hazards, uneven distribution of medical resources, and substantial population movement. Identifying factors associated with disaster preparedness in these settings may help inform workforce development, training priorities, and preparedness planning.

Disaster preparedness has become a major topic in disaster nursing research. In China, researchers have examined disaster preparedness among emergency nurses [10], nursing students [11], and male nurses [12], primarily through cross-sectional designs. However, these studies have mainly been conducted in inland provinces such as Sichuan and Henan, and evidence from border-area settings remains limited. In addition, researchers have often examined influencing factors separately, with less attention to the relative importance of multiple variables considered simultaneously. Recent international evidence has continued to identify disaster preparedness among nurses as an ongoing priority, particularly in disaster-prone and resource-constrained settings [13]. A systematic review further showed that nurses in high-risk countries experience important gaps in disaster-related knowledge and preparedness, underscoring the need for more context-specific evidence to guide workforce development and preparedness planning [14].

An increasing body of research has also suggested that disaster preparedness is related not only to technical knowledge and training but also to psychological and professional factors. In particular, resilience has been associated with healthcare workers' ability to cope with stress, uncertainty, and emergency demands, while professional mission may shape commitment, responsibility, and motivation in disaster response. These factors are especially relevant in high-pressure settings where nurses may need to sustain performance under conditions of uncertainty and limited resources. Studies have further suggested that nurses' disaster preparedness is linked to psychological resilience, disaster literacy, and organizational preparedness support [14]–[16]. For example, research has highlighted associations between preparedness and resilience among nurses, while qualitative evidence has emphasized the importance of strengthening disaster literacy and training capacity in nursing practice [14],[15]. Moreover, organizational and nursing management preparedness has also been recognized as an important contextual factor shaping disaster response capacity [16].

The random forest algorithm [17] is a machine-learning algorithm that can be used to rank the relative importance of variables and may therefore complement conventional statistical approaches when the aim is to identify priority correlates. Guided by the iceberg model, our study focused on resilience and sense of professional mission as potential correlates of disaster preparedness among nurses working in disaster-prone border areas of Southwest China. We aimed to examine the level of nurses' disaster preparedness in this setting and to identify priority correlates that may inform disaster nursing training and workforce preparedness strategies.

## 2. Methods

### 2.1. Study design

A multicenter cross-sectional survey was conducted to examine disaster preparedness among nurses working in disaster-prone border areas of Southwest China. The study was reported in accordance with the Strengthening the Reporting of Observational Studies in Epidemiology (STROBE) guidelines.

### 2.2. Samples and participants

The study was conducted in disaster-prone border areas of Southwest China. This region is characterized by frequent exposure to natural hazards, substantial population mobility, and relatively constrained health resources compared with many inland areas.

To improve representativeness within this border-area context, we sampled multiple prefectures and hospitals across development strata and hospital levels. From March 2023 to December 2023, a questionnaire survey was conducted among nurses working in disaster-prone border areas of Southwest China using a multistage stratified cluster sampling method.

In Stage 1, according to the gross domestic product (GDP) of each prefecture-level area in Yunnan Province in 2021 [18], the study area was divided into three strata: developed, subdeveloped, and underdeveloped. One prefecture-level area was randomly selected from each stratum, yielding three study sites: Baoshan City, Xishuangbanna Dai Autonomous Prefecture, and Nujiang Lisu Autonomous Prefecture.

In Stage 2, hospitals were selected using stratified sampling within each selected site, including one tertiary hospital, two secondary hospitals, and three primary hospitals, resulting in six hospitals for each site.

In Stage 3, cluster sampling was conducted within the selected hospitals. Departments were used as the primary sampling units in secondary and tertiary hospitals, whereas hospitals were used as the primary sampling units in primary hospitals.

Eligible participants were nurses who met all of the following criteria: (1) Held a valid Chinese nurse practice certificate and were registered as active nurses; (2) had worked in their current hospital for at least 1 year; and (3) voluntarily agreed to participate in the study. Nurses were excluded if they were (1) not on duty during the survey period or (2) temporarily not working in their registered hospital during the survey period.

According to the sample size formula for cross-sectional studies and with reference to the study of Wang [19], the standard deviation of the total disaster preparedness score was 40.80, µ = 1.96, δ = 6, and α = 0.05. The required sample size was estimated to be 178 participants in each stratum. After allowing for 10% invalid questionnaires, 196 participants were required in each stratum. Considering a design effect of 2 and three geographic strata, the minimum required sample size was calculated to be 1176.

In the actual survey, the multistage stratified cluster sampling strategy yielded a larger number of eligible nurses than the minimum required sample size. After questionnaire retrieval and data quality screening, 1366 valid questionnaires were retained for analysis.

### 2.3. Ethical consideration

This study was approved by the Medical Ethics Committee of the hospital (No. DFY20230620001). All procedures involving human participants were conducted in accordance with the ethical standards of the institutional research committee and the Declaration of Helsinki.

The study followed the principles of voluntary participation, anonymity, confidentiality, and non-maleficence. Informed consent was obtained from all participants before data collection. Completion and submission of the anonymous questionnaire were considered consent to participate. Before participation, all potential participants were informed of the purpose of the study, the voluntary nature of participation, and their right to decline participation or withdraw at any time without penalty. During data analysis, all data were anonymized and used only for research purposes.

### 2.4. Measurements

#### 2.4.1. Participant demographic and work-related characteristics

A structured questionnaire was used to collect participants' demographic and work-related characteristics, including gender, age, ethnicity, educational background, years of work experience, professional title, department, hospital level, disaster relief experience, and disaster-related training.

#### 2.4.2. The Chinese version of the disaster preparedness evaluation tool

Disaster preparedness was assessed using the Chinese version of the Disaster Preparedness Evaluation Tool. The original instrument was developed by Tichy and Bond [20] and later translated and adapted into Chinese by Li [21]. The scale contains 45 items across three dimensions: disaster preparedness knowledge, disaster response, and post-disaster management. Each item is rated on a 6-point Likert scale ranging from 1 (“strongly disagree”) to 6 (“strongly agree”), yielding a total score ranging from 45 to 270, with higher scores indicating better disaster preparedness.

According to other studies, a mean item score of 1.00–2.99 indicates poor disaster preparedness, 3.00–4.99 indicates moderate preparedness, and 5.00–6.00 indicates good preparedness. The Chinese version has been used in related studies and has demonstrated acceptable reliability. In the original Chinese validation study, the total Cronbach's α coefficient was reported as 0.865 [21]. In this study, the Cronbach's α coefficient was 0.975, indicating very high internal consistency.

#### 2.4.3. Resilience scale of medical staff

Resilience was measured using the Resilience Scale of Medical Staff developed by Zhu et al. [22]. The scale consists of 18 items covering four dimensions: decision coping, interpersonal connection, rational thinking, and flexibility and adaptability. Each item is rated on a 5-point Likert scale from 1 (“completely inconsistent”) to 5 (“completely consistent”), with total scores ranging from 18 to 90. Higher scores indicate higher levels of resilience. The reported Cronbach's α coefficient of the original scale was 0.880 [22]. In this study, the Cronbach's α coefficient was 0.951.

#### 2.4.4. Sense of professional mission scale

Sense of professional mission was measured using the Professional Mission Scale developed by Zhang [23] based on the Professional Mission Questionnaire proposed by Dobrow et al. [24] and adapted to the Chinese cultural context. The scale includes 11 items across three dimensions: altruistic contribution, orientation, and meaning and value. Items are rated on a 5-point Likert scale from 1 (“completely inconsistent”) to 5 (“completely consistent”), with total scores ranging from 11 to 55. Higher scores indicate a stronger sense of professional mission. The reported Cronbach's α coefficient of the scale was 0.746 [23]. In this study, the Cronbach's α coefficient was 0.919.

### 2.5. Data collection

Data were collected using online and paper-based questionnaires between March 2023 and December 2023. For the online survey, a self-administered questionnaire was developed using the Questionnaire Star platform and distributed through designated nursing administrators or unit heads in participating hospitals, who forwarded the survey link to eligible nurses. For the paper-based survey, questionnaires were distributed on site by the researcher or trained investigators after obtaining permission from the target units, and completed questionnaires were collected immediately upon completion.

Before participation, all potential participants were informed of the purpose of the study, the voluntary nature of participation, the anonymous and confidential handling of the survey, and their right to decline participation or withdraw at any time without penalty. After collection, all returned questionnaires were screened for data quality. Questionnaires with substantial missing data or obvious logical inconsistencies were excluded from the final analysis. Because the survey was administered through a coordinated site-based distribution and collection process rather than open online recruitment, the number of questionnaires included in the final analysis was determined after prespecified data quality screening. Only questionnaires that met these criteria were retained for analysis.

### 2.6. Data analysis

All data were analyzed using SPSS 29.0 and RStudio version 4.3.2. A two-sided P value < 0.05 was considered statistically significant. Categorical variables were summarized using frequencies and percentages, and continuous variables were described using means and standard deviations. Group differences in disaster preparedness scores were examined using independent-samples t tests or one-way analysis of variance, as appropriate. Pearson correlation analysis was used to examine bivariate associations among disaster preparedness, resilience, and sense of professional mission.

To further identify predictors of disaster preparedness, variables with statistical significance in the univariate or correlation analyses were first entered into a least absolute shrinkage and selection operator (LASSO) procedure for variable screening. Variables retained by LASSO were then entered into a multivariable linear regression model with disaster preparedness total score as the dependent variable to estimate independent associations. Collinearity diagnostics were examined during model evaluation, including tolerance and variance inflation factor (VIF). Residual normality, homoscedasticity, and independence were also assessed using residual plots, normal probability plots, and the Durbin–Watson statistic.

In addition, random forest analysis was conducted to rank the relative importance of factors associated with disaster preparedness. The disaster preparedness total score was treated as the dependent variable, and variables showing statistical significance in the univariate or correlation analyses were entered into the model as predictors. Variable importance was evaluated using the percentage increase in mean squared error (%IncMSE), with higher values indicating greater relative importance.

The random forest model was implemented using the randomForest package in R with the default settings for regression trees (ntree = 500; mtry = 4). Variable importance was derived from the increase in out-of-bag mean squared error after variable permutation. The random forest model was used as a complementary analytic approach because its primary purpose in this study was to compare the relative importance of multiple variables and identify priority correlates rather than to estimate causal effects.

## 3. Results

A total of 1394 questionnaires were distributed through the coordinated site-based distribution and collection process. After data quality screening, 28 questionnaires were excluded because of substantial missing data or obvious logical inconsistencies. Finally, 1366 questionnaires met the prespecified data quality criteria and were included in the final analysis.

### 3.1. Characteristics of participants

Among the 1366 nurses included in the analysis, 1161 (85.0%) were female and 205 (15.0%) were male. The largest age group was 26–35 years (50.2%), followed by ≥36 years (31.1%) and ≤25 years (18.7%). Regarding ethnicity, 48.7% were Han, and 51.3% belonged to ethnic minority groups. In terms of educational background, 49.1% had a bachelor's degree or above.

With respect to work-related characteristics, 30.4% had worked for 11–15 years, and 36.1% held the title of nurse. Emergency department nurses accounted for 25.5% of the sample, and 65.6% worked in tertiary hospitals. In addition, 44.9% of participants reported previous disaster relief experience, and 67.1% had received disaster-related training. Detailed participant characteristics are presented in Table 1.

**Table 1.** Demographic characteristics of surveyed nurses (n = 1366).

Variables	category	n	%
Gender	Male	205	15.0
	Female	1161	85.0
Age (Years)	≤25	255	18.7
	26–35	686	50.2
	≥36	425	31.1
Ethnicity	Han nationality	665	48.7
	Dai nationality	84	6.1
	Bai nationality	233	17.1
	Yi nationality	377	27.6
	Other	7	0.5
Educational background	Technical secondary school and below	116	8.5
	Junior college	580	42.4
	Undergraduate	665	48.7
	Postgraduate and above	5	0.4
Years of work experience	<5	299	21.9
	5–10	257	18.8
	11–15	415	30.4
	16–20	227	16.6
	>20	168	12.3
Professional title	Nurse	493	36.1
	Senior nurse	460	33.7
	Nurse-in-charge	274	20.0
	Associate chief nurse and above	139	10.2
Specialty	Emergency	348	25.5
	Intensive care unit	85	6.2
	Internal medicine	232	17.0
	Surgery	299	21.9
	Outpatient service	346	25.3
	Other	56	4.1
Hospital level	Tertiary hospital	896	65.6
	Secondary hospital	356	26.1
	First-class hospital	114	8.3
Disaster experience	Yes	614	44.9
	No	752	55.1
Disaster training	Yes	916	67.1
	No	450	32.9

**Table 2.** Comparison of nurses' disaster preparedness in general data (n = 1366).

Variables	category	Mean	SD	t/F	P
Gender	Male	190.34	32.47	3.595^a^	<0.001
	Female	180.91	45.04		
Age (Years)	≤25	158.05	37.64	80.309^b^	<0.001
	26–35	180.90	40.56		
	≥36	199.18	44.10		
Ethnicity	Han nationality	190.99	44.15	15.875^b^	<0.001
	Dai nationality	171.92	41.22		
	Bai nationality	171.18	38.75		
	Yi nationality	175.54	42.58		
	Other	219.71	22.45		
Educational background	Technical secondary school and below	171.22	39.62	19.256^b^	<0.001
	Junior college	174.45	42.19		
	Undergraduate	191.26	43.59		
	Postgraduate and above	163.80	38.49		
Years of work experience	<5	161.36	39.23	39.112^b^	<0.001
	5–10	175.61	41.41		
	11–15	185.61	40.69		
	16–20	195.30	41.22		
	>20	204.24	45.79		
Professional title	Nurse	160.01	36.28	84.073^b^	<0.001
	Senior nurse	191.73	40.72		
	Nurse-in-charge	195.00	41.95		
	Associate chief nurse and above	205.33	45.89		
Specialty	Emergency	174.82	42.55	11.147^b^	<0.001
	Intensive care unit	184.35	41.53		
	Internal medicine	188.80	46.78		
	Surgery	181.29	42.52		
	Outpatient service	180.19	41.59		
	Other	217.71	35.09		
Hospital level	Tertiary hospital	184.63	43.54	4.776^b^	0.009
	Secondary hospital	179.60	43.69		
	First-class hospital	172.68	41.18		
Disaster experience	Yes	191.51	42.04	7.201^a^	<0.001
	No	174.82	43.27		
Disaster training	Yes	195.53	42.37	19.534^a^	<0.001
	No	155.43	31.85		

Note: ^a^ is t value; ^b^ is the F value.

### 3.2. Disaster preparedness scores of nurses

The mean total disaster preparedness score among the 1366 nurses was 182.32 ± 43.51, corresponding to a mean item score of 4.05 ± 0.97, which indicated a moderate level of disaster preparedness. Across the three dimensions, the highest mean item score was observed for disaster response (4.09 ± 1.13), followed by post-disaster management (4.06 ± 1.19) and disaster preparedness knowledge (4.03 ± 1.14).

### 3.3. Univariate analysis of disaster preparedness

Univariate analyses showed statistically significant differences in disaster preparedness scores according to gender, age, ethnicity, educational background, years of work experience, professional title, specialty, hospital level, disaster relief experience, and disaster-related training (all P < 0.05; Table 2). Specifically, higher disaster preparedness scores were observed among male nurses, older nurses, those with more years of work experience, those with higher professional titles, and those who had disaster relief experience or disaster-related training. Significant differences were also observed across ethnic groups, educational background categories, specialties, and hospital levels. Detailed results are presented in Table 2.

### 3.4. Correlation analysis of disaster preparedness, resilience, and sense of professional mission

The mean resilience score of the nurses was 58.66 ± 19.45, and the mean sense of professional mission score was 35.50 ± 11.99. Pearson correlation analysis showed that disaster preparedness was positively correlated with resilience (r = 0.729, P < 0.01) and with sense of professional mission (r = 0.701, P < 0.01). These findings indicate that nurses with higher levels of resilience and professional mission tended to report higher levels of disaster preparedness.

### 3.5. Random forest analysis of factors associated with disaster preparedness

To compare the relative importance of factors associated with disaster preparedness, a random forest model was constructed using disaster preparedness total score as the dependent variable. Twelve variables that showed statistical significance in the univariate or correlation analyses were entered into the model as predictors. Variable importance was evaluated using the percentage increase in mean squared error (%IncMSE) [25], with higher values indicating greater relative importance in predicting disaster preparedness. The model was implemented using the RandomForest package in R with the default settings for regression trees (ntree = 500; mtry = 4).

The random forest analysis showed that resilience had the highest importance score (%IncMSE = 75.226), followed by gender (%IncMSE = 60.480) and sense of professional mission (%IncMSE = 57.707). Other variables, in descending order of importance, were disaster-related training (%IncMSE = 28.639), professional title (%IncMSE = 26.601), years of work experience (%IncMSE = 24.569), age (%IncMSE = 17.049), disaster relief experience (%IncMSE = 12.537), educational background (%IncMSE = 10.597), specialty (%IncMSE = 5.741), hospital level (%IncMSE = 1.450), and ethnicity (%IncMSE = 1.328). Detailed importance scores are presented in Table 3 and Figure 1.

To further refine the candidate predictors for regression modeling, LASSO regression was performed after the random forest ranking. When the penalty parameter λ was 0.2756626, model error was minimized, and 10 variables were retained. These variables were resilience, gender, sense of professional mission, disaster-related training, professional title, years of work experience, age, disaster relief experience, educational background, and specialty.

These results suggest that resilience, gender, and sense of professional mission were the most prominent correlates of disaster preparedness in this sample. However, the random forest results should be interpreted as reflecting relative variable importance rather than independent or causal effects.

**Table 3.** Importance score of independent variables

Variable	%IncMSE
Gender	60.480
Age	17.049
Ethnicity	1.328
Educational background	10.597
Years of work experience	24.569
Professional title	26.601
Specialty	5.741
Hospital level	1.450
Disaster experience	12.537
Disaster training	28.639
Resilience	75.226
Professional mission	57.707

**Figure 1.**
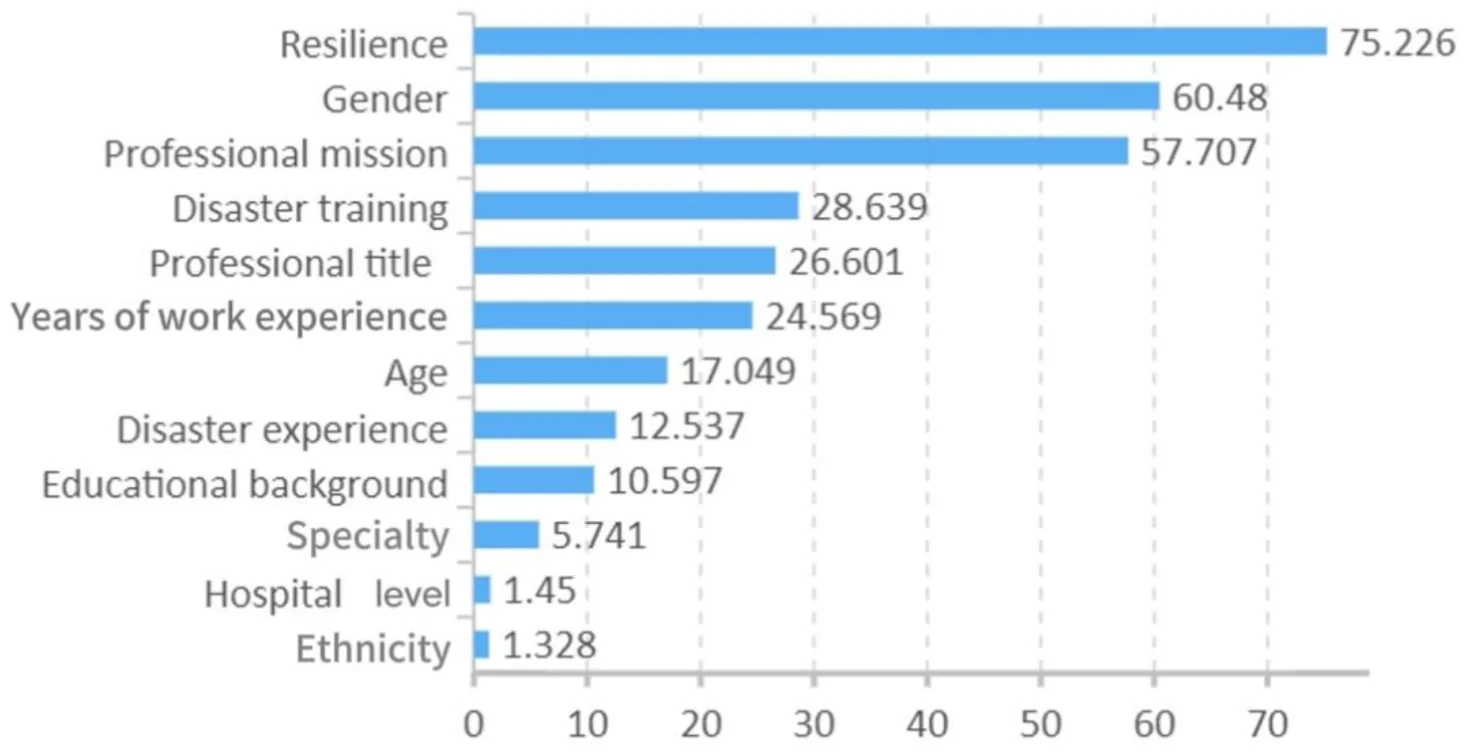
Ranking of variable importance in the random forest model for disaster preparedness.

### 3.6. Multivariable linear regression analysis of disaster preparedness

The 10 variables retained by the LASSO procedure were entered into a multivariable linear regression model with disaster preparedness total score as the dependent variable. The final model was statistically significant (F = 217.10, P < 0.001) and explained 61.6% of the variance in disaster preparedness (R² = 0.616; adjusted R² = 0.613).

In the multivariable model, resilience (B = 0.936, P < 0.001), sense of professional mission (B = 1.086, P < 0.001), gender (B = −18.721, P < 0.001), and disaster-related training (B = −12.455, P < 0.001) remained significant predictors of disaster preparedness. By contrast, professional title, years of work experience, educational background, specialty, age, and disaster relief experience were not statistically significant after mutual adjustment (all P > 0.05).

Collinearity diagnostics indicated no evidence of severe multicollinearity based on the prespecified criteria, with tolerance values ranging from 0.107 to 0.980 and VIF values ranging from 1.020 to 9.303. Residual diagnostics showed that the histogram and normal P–P plot were generally consistent with normality, the residual scatterplot supported homoscedasticity, and the Durbin–Watson statistic was 2.068, indicating independence of residuals. These results suggest that the assumptions of the linear regression model were adequately met.

## 4. Discussion

In this study, disaster preparedness among nurses working in disaster-prone border areas of Southwest China was examined, and the results indicated a moderate level of preparedness. The random forest analysis ranked resilience, gender, and sense of professional mission as the most prominent correlates of disaster preparedness. From a public health perspective, these findings provide useful evidence for prioritizing workforce preparedness strategies in border areas of Southwest China. In particular, disaster preparedness planning may benefit from attention not only to technical training, but also to psychological resilience and professional commitment.

### 4.1. Status of disaster preparedness among nurses

In this study, nurses working in disaster-prone border areas of Southwest China showed a moderate level of disaster preparedness. The mean total score and mean item score were slightly higher than those reported in some previous studies using the same instrument [26], which may reflect the increasing attention given to emergency preparedness and disaster-related training in recent years, particularly following major public health emergencies and disaster events. Furthermore, the preparedness level observed in this study was lower than that reported in some other regions of China [27], which may be related to differences in geographic context, health resource distribution, and local training capacity.

Among the three dimensions of disaster preparedness, disaster response had the highest mean score, whereas disaster preparedness knowledge had the lowest. This pattern suggests that nurses may be relatively more familiar with practical response demands than with systematic disaster-related knowledge. One possible explanation is that, although emergency response has received increasing attention in clinical practice, access to structured disaster-preparedness education and specialized training may be uneven across institutions and regions, particularly in resource-constrained settings [28]. These findings indicate that border areas may benefit from strengthening knowledge-oriented and competency-based disaster preparedness training in addition to practical emergency response drills.

### 4.2. Factors associated with disaster preparedness

#### 4.2.1. Resilience and disaster preparedness

This study showed that resilience was the most prominent correlate of disaster preparedness in the random forest analysis, and disaster preparedness was positively correlated with resilience. This finding is consistent with other studies [29], suggesting that resilience is closely related to healthcare workers' ability to respond to pressure, uncertainty, and crisis situations. Nurses with higher levels of resilience may be better able to maintain emotional stability, adapt to rapidly changing situations, and sustain effective functioning in emergency contexts. From a practical perspective, this finding highlights the potential value of psychological support, resilience-building interventions, and supportive organizational environments in disaster preparedness planning.

#### 4.2.2. Gender and disaster preparedness

Gender also emerged as an important correlate of disaster preparedness in the random forest model, with male nurses showing higher preparedness scores in the univariate analysis. This finding should be interpreted cautiously and not as evidence of inherent gender-based differences in disaster competence. A more plausible explanation is that gender differences in preparedness scores may reflect differences in role allocation, disaster-related experience, training opportunities, or task assignment within healthcare settings. In practice, this result underscores the importance of ensuring equitable access to disaster preparedness training and emergency participation opportunities across staff groups.

#### 4.2.3. Sense of professional mission and disaster preparedness

Sense of professional mission was another prominent correlate of disaster preparedness in this study. Nurses with a stronger sense of professional mission may be more likely to maintain commitment, responsibility, and engagement in disaster-related work [30]. This may be particularly relevant in emergency contexts where sustained motivation, teamwork, and professional responsibility are essential. From a workforce development perspective, the finding suggests that disaster preparedness strategies may benefit not only from technical training but also from efforts to foster professional identity, meaning, and commitment within nursing teams [31].

### 4.3. Contextual interpretation and transferability

The findings of this study should be interpreted within the context of border areas of Southwest China. This setting is characterized by recurrent disaster exposure, population mobility, and uneven health resource distribution, all of which may shape nurses' preparedness needs. However, we included only nurses working on the Chinese side of the border and did not directly assess cross-border healthcare coordination, transnational workforce mobility, or other specific cross-border contextual mechanisms. Therefore, the findings should not be interpreted as representing nurses on both sides of an international border or as directly demonstrating the effects of cross-border contextual factors.

Furthermore, some contextual features observed in this study, such as disaster vulnerability, resource constraints, and service pressure in border areas, may also be relevant to other high-risk or resource-limited settings. In this sense, the study may offer useful reference points for disaster preparedness planning in broadly comparable contexts. Nevertheless, the applicability of these findings to other geographic, institutional, or national settings should be considered cautiously and requires further validation in multi-site and cross-context studies.

## 5. Limitations

This study has several limitations. First, this cross-sectional survey was conducted in border areas of Southwest China and included only nurses working on the Chinese side of the border. Therefore, the findings should be interpreted within this geographic and institutional context. Because we did not include nurses from Myanmar or directly assess cross-border healthcare coordination, transnational workforce mobility, or other cross-border contextual mechanisms, the results should not be interpreted as representing nurses on both sides of the border.

Second, potential selection bias cannot be excluded. Although we used a multistage stratified cluster sampling strategy, participation was voluntary, and the survey was distributed through a coordinated site-based process. Nurses who were more available, more engaged, or more interested in disaster preparedness may have been more likely to participate. This may have led to an overestimation of disaster preparedness levels and may limit the representativeness of the findings.

Third, information bias may have occurred because disaster preparedness, resilience, sense of professional mission, and related experiences were measured using self-reported questionnaires. Participants may have overreported socially desirable responses, such as preparedness, training experience, or professional commitment. As a result, the levels of disaster preparedness and its associations with psychological and professional variables may have been overestimated.

Fourth, residual confounding cannot be ruled out. Although multiple demographic and work-related variables were included in the regression model, some potentially important factors were not measured, such as organizational disaster preparedness support, quality and frequency of disaster training, previous disaster exposure intensity, staffing levels, and institutional emergency management resources. These unmeasured factors may have influenced nurses' preparedness and the identified correlates, which means that the observed associations should not be interpreted as independent causal effects.

Fifth, the use of online and paper-based questionnaires may have introduced potential mode effects related to response context, completion behavior, and data quality. Such mode effects may have affected the comparability of responses between participants completing the survey online and those completing the paper-based questionnaire.

In addition, the very high Cronbach's α coefficient of the Disaster Preparedness Evaluation Tool in this sample suggests excellent internal consistency, although it may also indicate some degree of overlap among scale items. Finally, because of the cross-sectional design, causal inferences cannot be made. The observed associations and random forest importance rankings should therefore be interpreted as exploratory and correlational rather than causal. Future multi-site studies with broader geographic coverage, more detailed contextual measures, and longitudinal or mixed-methods designs are needed to further validate these findings.

## 6. Conclusions

Disaster preparedness among nurses working in disaster-prone border areas of Southwest China was moderate in this study. Resilience, gender, and sense of professional mission emerged as prominent correlates of disaster preparedness, suggesting that disaster preparedness planning may benefit from attention not only to technical training but also to psychological and professional dimensions of workforce development.

From a practical perspective, the findings suggest that disaster preparedness in border areas may be strengthened by combining emergency response training with efforts to support resilience, reinforce professional commitment, and expand equitable access to preparedness-related learning opportunities. Furthermore, these findings should be interpreted within the context of border areas of Southwest China and should not be generalized uncritically to other national or cross-border settings.

## Use of AI tools declaration

The authors declare they have not used Artificial Intelligence (AI) tools in the creation of this article.
